# Clinical outcome of ≥2% circulating tumor cells in newly diagnosed multiple myeloma: insights from a multicenter study

**DOI:** 10.1080/07853890.2025.2496796

**Published:** 2025-04-30

**Authors:** Dong Liang, Yurong Yan, Shenrui Bai, Weiling Xu, Qiaoli Wang, Demei Feng, Yuying Bu, Min Zeng, Xiaomiao Nie, Yuan Feng, Xiaoqin Chen, Zhongjun Xia, Yang Liang, Fengyan Jin, Hua Wang

**Affiliations:** a State Key Laboratory of Oncology in South China, Guangdong Provincial Clinical Research Center for Cancer, Sun Yat-Sen University Cancer Center, Guangzhou, China; b Hematology Department, First Hospital of Jilin University, Changchun, China; c Radiology Department, First Hospital of Jilin University, Changchun, China

**Keywords:** Circulating tumor cells, clinical outcome, multiple myeloma

## Abstract

**Purpose:**

Previous studies have shown that ≥2% circulating tumor cells (CTCs) in multiple myeloma are associated with a prognosis similar to primary plasma cell leukemia. This study aims to examine this ultra-high-risk patient subset and evaluate their clinical outcomes in a real-world clinical setting.

**Methods:**

We included 1,056 newly diagnosed multiple myeloma patients treated with novel agents. CTCs levels were determined *via* morphological assessment on peripheral blood smears, using a 2% cutoff to stratify patients into <2% and ≥2% CTCs groups. We then evaluated clinical outcomes across these groups.

**Results:**

Patients with ≥2% CTCs constitute an ultra-high-risk subgroup, with outcomes resembling those of primary plasma cell leukemia. Survival outcomes improved for patients receiving daratumumab-based quadruplet therapy. Single autologous stem cell transplantation (ASCT) partially improved outcomes for patients with ≥2% CTCs. Achieving complete remission (CR) after induction treatment did not confer a better prognosis for this population. Furthermore, one high-risk cytogenetic abnormality (HRA) worsened outcomes in the <2% CTC group, while ≥2 HRA were associated with poorer outcomes in the ≥2% CTC group. Concurrent 1q21+ and other HRA further conferred a worse prognosis. In de novo extramedullary extraosseous (EME) multiple myeloma, defined as patients presenting with soft tissue or visceral plasmacytomas not connected to bone at initial diagnosis, ≥2% CTCs remained a strong predictor of poor prognosis.

**Conclusion:**

Our study suggests that patients with ≥2% CTCs represent a distinct ultra-high-risk subgroup in multiple myeloma and warrant separate consideration. VRD, IRD, DVRD, and DRD were reliable choices as frontline therapies for patients with <2% CTCs. Daratumumab-based quadruplet therapy may be a promising option for patients with ≥2% CTCs. Further research should continue to explore this specific aspect in greater depth.

## 1. Introduction

Multiple myeloma is the second most common hematological malignancy, characterized by the accumulation of plasma cells in the bone marrow [1,2]. These plasma cells can migrate into peripheral blood, indicating the disease’s progression. The presence of plasma cells in the peripheral blood is considered a prognostic marker for poor outcomes in multiple myeloma patients [3–6]. In a recent meta-analysis that included 5,637 myeloma patients, circulating plasma cells were shown to be a reliable predictive biomarker for poor prognosis in multiple myeloma [7]. Both flow cytometry and morphological assessment by peripheral blood smear have established cutoff values for CTCs that can stratify patients into good and poor prognosis groups [8,9].

Plasma cell leukemia, historically defined by the presence of more than 20% CTCs, is associated with an extremely poor prognosis [10–12]. Recently, the International Myeloma Working Group revised the CTCs cutoff for primary plasma cell leukemia to 5% [13]. A multicenter study conducted across five university hospitals in Catalonia found that patients with 5–20% CTCs exhibited a prognosis comparable to those with more than 20% CTCs [14]. A study from the Mayo Clinic supported these findings, revealing that patients with 5–20% CTCs had a prognosis similar to those with more than 20% CTCs [15]. Notably, having more than 5% CTCs was associated with an extremely poor prognosis. These findings provide a strong rationale for establishing the threshold of 5% CTCs as a criterion for diagnosing primary plasma cell leukemia.

Furthermore, a multicenter study recommended using a 2% CTCs threshold, suggesting it correlates with a plasma cell leukemia-like prognosis and highlights the highly aggressive nature of circulating plasma cells [16]. In this study, we aim to evaluate the prognostic value of this new 2% plasma cell cutoff in the era of novel agents, using data from a multicenter cohort.

## 2. Materials and methods

### 2.1. Patient population

Clinical data from Sun Yat-sen University Cancer Center and the First Hospital of Jilin University were retrospectively analyzed for patients newly diagnosed with multiple myeloma between January 2016 and October 2023, with a final follow-up on September 1, 2024. The Institutional Ethical Review Board of Sun Yat-sen University Cancer Center approved the anonymized data analysis and waived informed consent. Rigorous procedures ensured data accuracy and completeness. The International Conference on Harmonization’s Good Clinical Practice standards and the 1964 Helsinki Declaration and its later revisions served as the foundation for the study’s methodology.

Inclusion criteria comprised newly diagnosed multiple myeloma (NDMM) patients from both centers. Exclusion criteria included patients who did not undergo PET/CT or MRI examinations, those lost to follow-up, and those with ≥20% circulating tumor cells (CTCs). The number of CTCs in this study was determined by morphological assessment on Wright-Giemsa-stained peripheral blood (PB) smears, with a minimum of 200 nucleated cells examined per smear.

Extramedullary disease (EMD) encompasses two distinct forms: extramedullary extraosseous (EME), characterized by soft tissue or visceral plasmacytomas not connected to bone, and extramedullary bone-related (EMB), identified as paraskeletal plasmacytomas. De novo EME referred to patients with soft tissue or visceral plasmacytoma not connected to the bone at initial diagnosis. EMB was identified as paraskeletal plasmacytoma. Patients with both EMB and EME were classified as EME. Patients initially diagnosed with non-EMD who later developed EMB remained classified as non-EMD. EMB and EME diagnoses were confirmed by PET/CT and biopsy. Non-EME included non-EMD and EMB. Both centers used the same inclusion and exclusion criteria across four multiple myeloma groups.

Bone marrow biopsies were performed on all patients before initial treatment. Bone marrow aspirations were sorted using CD138 magnetic beads to purify plasma cells, which were then analyzed by FISH (Fluorescence *In Situ* Hybridization) for chromosomal abnormalities, including (del) (13q14) (13q-), del(17p) (17p-), t(4;14), t(11;14), t(14;16), and 1q21 gain/amplification (1q21+). High-risk cytogenetic abnormalities (HRA) included 1q21+, t(4;14), t(14;16), and del(17p). Patients with at least one HRA were classified as high-risk, while those without HRA were classified as standard-risk. ‘Double-hit’ patients had two HRA. Due to the limited number of patients with ≥3 HRA, patients with 2 HRA and those with ≥3 HRA were classified into those having ≥2 HRA in our study. A P-value of less than 0.05 was considered statistically significant.

### 2.2. Statistical analysis

Baseline characteristics were compared between treatment groups using Fisher’s exact test and the Chi-squared test for categorical variables, and the Wilcoxon rank-sum test or Student’s t-test for continuous variables. Progression free survival (PFS) was the time from induction initiation to death or progression, while overall survival (OS) was defined as the time from induction initiation to death from any cause. Kaplan-Meier curves were used to visualize PFS and OS across different groups. Follow-up was calculated using reverse Kaplan-Meier method. The multivariate model was employed to evaluate the impact of selected variables—including cytogenetic risk stratification, ISS stage, LDH level, and CTCs level—on survival outcomes in patients with multiple myeloma. Pearson’s and Spearman’s rank tests were employed for correlation analysis. All analyses were conducted using CRAN R Version 4.3.2 (The R Foundation for Statistical Computing, Vienna, Austria).

## 3. Results

### 3.1. Baseline characteristics

In this study, a total of 1,056 patients were included. The median follow-up period for the entire cohort was 27 months (95% CI: 25–28). The median follow-up was 28 months (95%CI: 24–53) for patients with ≥2% CTCs and 26 months (95%CI: 24–28) for patients with <2% CTCs. Baseline characteristics recorded at initial diagnosis included induction treatment regimen, sex, age, extramedullary extraosseous (EME) category, immunoglobulin type, Eastern Cooperative Oncology Group (ECOG) score, transplantation status, cytogenetic risk stratification, 17p deletion, t(4;14), t(14;16), 13q deletion, t(11;14), 1q21+, international staging system (ISS) stage, revised international staging system (RISS) stage, bone marrow plasma cell percentage (BMPC%), lactate dehydrogenase (LDH) levels, creatinine (Cr), calcium (Ca), and hemoglobin (HGB) levels. Notably, patients with ≥2% CTCs had a higher proportion of high-risk features, including 1q21+, advanced ISS and RISS stages, elevated BMPC%, LDH, Cr, and Ca levels, and lower HGB levels compared to those with <2% CTCs. These differences in baseline variables suggest a more aggressive disease course for patients with ≥2% CTCs compared to those with <2% CTCs (Table 1).

**Table 1.** Characteristics at baseline.

	All patients	<2% CTCs	≥2% CTCs	
n	1056	926	49	*p* Value
**Drug (%)**				
PI plus IMiDs	473 (48.0)	433 (49.8)	17 (36.2)	0.181
CD38-based	144 (14.6)	128 (14.7)	8 (17.0)	
PI or IMiDs	369 (37.4)	309 (35.5)	22 (46.8)	
Unknown	70	56	2	
**Sex (%)**				
Female	466 (44.1)	409 (44.2)	19 (38.8)	0.553
Male	590 (55.9)	517 (55.8)	30 (61.2)	
**Age**				
≥65	370 (35.0)	323 (34.9)	16 (32.7)	0.869
<65	686 (65.0)	603 (65.1)	33 (67.3)	
**MM type**				
De novo EME	137 (13.0)	119 (12.9)	6 (12.2)	1
Non-EME	919 (87.0)	807 (87.1)	43 (87.8)	
**MM type**				
IgA	220 (21.2)	183 (20.0)	14 (29.2)	0.245
IgG	464 (44.7)	416 (45.5)	16 (33.3)	
IgD	45 (4.3)	37 (4.0)	4 (8.3)	
Light chain only	194 (18.7)	177 (19.4)	11 (22.9)	
Non-secretory	110 (10.6)	96 (10.5)	3 (6.2)	
Biclonal	5 (0.5)	5 (0.5)	0 (0.0)	
Unknown	18	12	1	
**ECOG score (%)**				
0–1	613 (58.1)	550 (59.5)	24 (49.0)	0.192
≥2	442 (41.9)	375 (40.5)	25 (51.0)	
Unknown	1	1	0	
**Transplantation (%**)				
Yes	216 (21.9)	199 (22.9)	8 (17.0)	0.45
No	770 (78.1)	671 (77.1)	39 (83.0)	
Unknown	70	56	2	
**Risk stratification (%)**				
High risk	359 (43.7)	312 (42.8)	26 (63.4)	0.015
Standard risk	462 (56.3)	417 (57.2)	15 (36.6)	
Unknown	235 (22.3)	197 (21.3)	8 (16.3)	
**13q-**	234 (30.0)	203 (29.3)	18 (43.9)	0.072
**t (11;14)**	78 (10.7)	62 (9.6)	7 (20.0)	0.09
**17p- (%)**	60 (7.3)	49 (6.7)	5 (12.2)	0.307
**t (4;14) (%)**	69 (9.0)	61 (8.9)	3 (8.6)	1
**t (14;16) (%)**	16 (2.1)	12 (1.8)	2 (5.7)	0.305
**1q21+ (%)**	319 (38.9)	279 (38.3)	24 (58.5)	0.016
**ISS stage (%)**				
1	251 (24.6)	236 (26.0)	3 (6.5)	<0.001
2	343 (33.6)	313 (34.5)	5 (10.9)	
3	428 (41.9)	357 (39.4)	38 (82.6)	
Unknown	34	20	3	
**RISS stage (%)**				
1	157 (17.6)	148 (18.8)	2 (4.8)	<0.001
2	587 (66.0)	527 (66.9)	19 (45.2)	
3	146 (16.4)	113 (14.3)	21 (50.0)	
Unknown	166	138	7	
**BMPC%**				<0.001
**Median (min-max)**	19.75 [0.0–99.0]	17.50 [0.00, 99.0]	54.00 [10.00, 93.5]	
**CTCs (%)**				
**Median (min-max)**	0 [0–12]	0 [0–1]	3 [2–12]	
≥2% CTCs	49 (5.0)	0	49	
<2% CTCs	926 (95.0)	926	0	
Unknown	81	0	0	
**LDH (%)**				<0.001
Median LDH—(IQR)	168 [134–216.8]	168 [134–214]	189.2 [135–293.9]	
≥250	167 (16.0)	134 (14.7)	17 (34.7)	
<250	874 (84.0)	779 (85.3)	32 (65.3)	
Unknown	15	13	0	
**Cr (%)**				0.008
Median Cr—(IQR)	79.5 [62.5–127.95]	78.4 [62.3–123]	114.9 [80.6–281.2]	
≥177	183 (17.4)	154 (16.7)	16 (32.7)	
<177	868 (82.6)	767 (83.3)	33 (67.3)	
Unknown	5	5	0	
**Ca (%)**				<0.001
Median Ca—(IQR)	2.26 [2.13–2.43]	2.26 [2.13–2.43]	2.44 [2.23–2.95]	
≥2.75	100 (9.5)	79 (8.6)	15 (30.6)	
<2.75	953 (90.5)	844 (91.4)	34 (69.4)	
Unknown	3	3	0	
**HGB (%)**				
Median HGB—(IQR)	97 [76–119]	99 [77–120]	77 [62–95]	
≥100	491 (46.7)	448 (48.5)	10 (20.4)	<0.001
<100	561 (53.3)	475 (51.5)	39 (79.6)	
Unknown	4	3	0	

ECOG: Score Eastern Cooperative Oncology Group Score; ISS stage: International Staging System; RISS stage: Revised International Staging System; BMPC%: bone marrow plasma cell percentage; LDH: lactate dehydrogenase; Cr: creatinine; Ca: calcium; HGB: hemoglobin; De novo EME: de novo extramedullary extraosseous; Non-EME: non extramedullary extraosseous.

### 3.2. Correlation between BMPC% and CTCs

**Figure 1.**
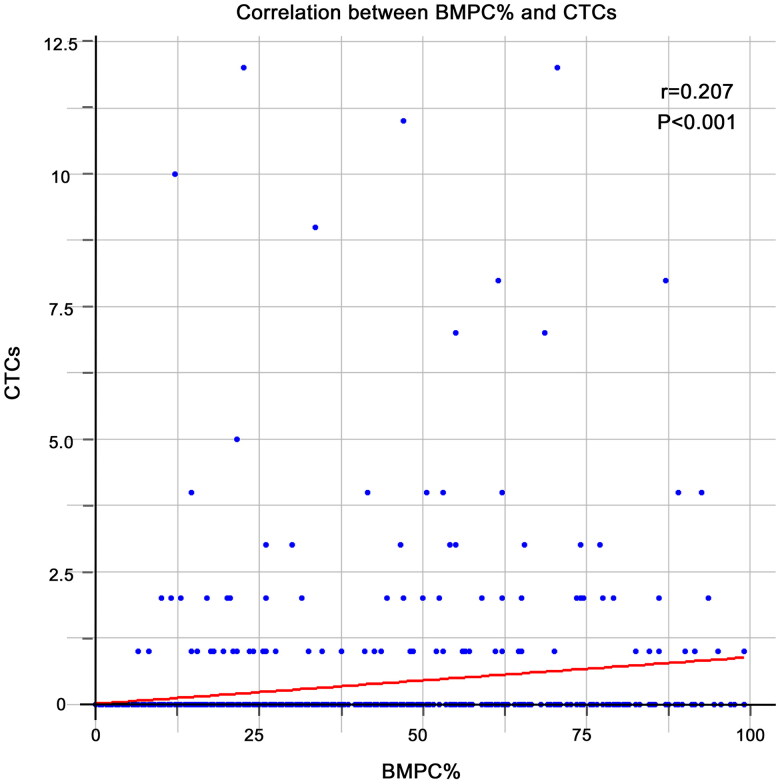
Correlation between bone marrow plasma cell percentage (BMPC%) and circulating tumor cells percentage.

The median BMPC% for the entire cohort was 19.75 (min-max: 0–99.0), with a median of 17.50 (min-max: 0–99.0) for patients with <2% CTCs and 54.00 (min-max: 10–93.50) for those with ≥2% CTCs. The median CTC percentage in the entire cohort was 0 (min-max: 0–12). For patients with <2% CTCs, the median CTC percentage was 0 (min-max: 0–1), whereas for those with ≥2% CTCs, the median CTC percentage was 3 (min-max: 2–12). A modest correlation was observed between BMPC% and CTCs (*p* = 0.21, *p* < 0.001) (Figure 1; Table 2).

**Table 2.** CTCs percentage distribution.

CTCs (%)	Overall cohort	De novo EME	Non-EME
Median [min-max]	0 [0–12%]	0 [0–7%]	0 [0–12%]
[0–1%] (number of patients)	926	119	807
[1–2%] (number of patients)	24	2	22
[2–3%] (number of patients)	8	1	7
[3–5%] (number of patients)	8	2	6
[5–20%] (number of patients)	9	1	8

CTCs: Circulating tumor cells; De novo EME: de novo extramedullary extraosseous; Non-EME: non extramedullary extraosseous.

### 3.3. ≥ 2% CTCs conferred a worse prognosis compared to <2% CTCs

**Figure 2.**
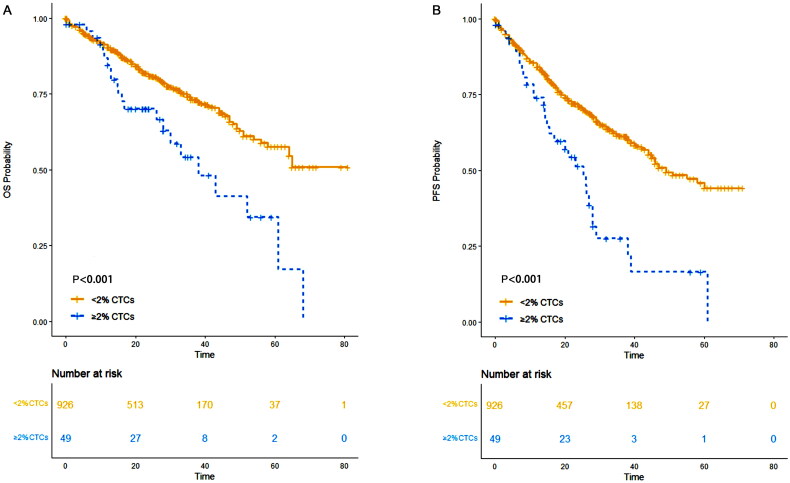
OS and PFS of patients with ≥2% CTCs and those with <2% CTCs. (A) OS; (B) PFS.

In our study, among the entire cohort, 49 patients had ≥2% CTCs and 926 patients had <2% CTCs. Patients with <2% CTCs demonstrated a five-year PFS of 44.2% (95% CI: 37.7%-51.9%) and a five-year OS of 57.6% (95% CI: 51.2%-64.8%). In contrast, patients with ≥2% CTCs had a five-year PFS of 16.6% (95% CI: 6.7%-41.1%) and a five-year OS of 34.4% (95% CI: 18.6%-63.6%). The median PFS was 49 months (95% CI: 45 months-not reached [NR]) for patients with <2% CTCs, compared to 25 months (95% CI: 16–29 months) for those with ≥2% CTCs. The median OS for patients with <2% CTCs was not reached (95% CI: 64 months-NR), while for those with ≥2% CTCs, the median OS was 38 months (95% CI: 28 months-NR). Patients with <2% CTCs exhibited significantly better OS (*p* < 0.001) and PFS (*p* < 0.001) compared to those with ≥2% CTCs (Figure 2).

### 3.4. 2–5% CTCs conferred a primary plasma cell leukemia like prognosis

**Figure 3.**
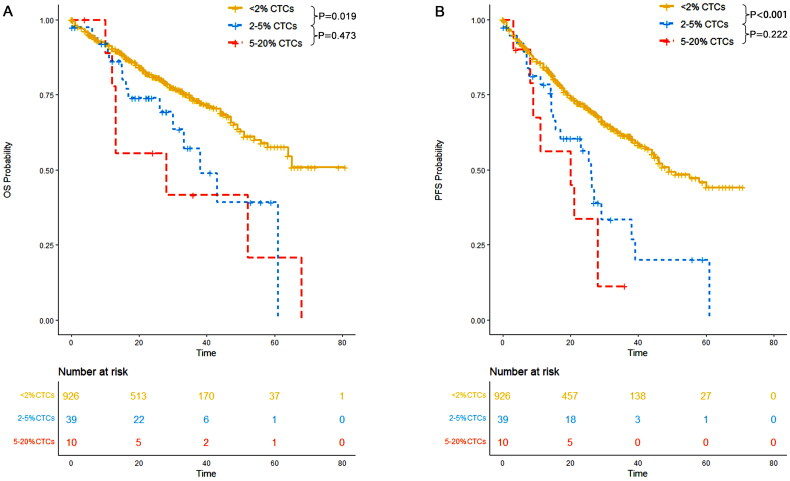
OS and PFS of patients with <2% CTCs, those with 2–5% CTCs and those with 5–20% CTCs. (A) OS; (B) PFS.

In our study, 39 patients had 2–5% CTCs, while 10 patients had ≥5% CTCs, which is defined as primary plasma cell leukemia. The median progression-free survival (PFS) was 26 months (95% CI: 16–39) for patients with 2–5% CTCs and 20 months (95% CI: 9-NR) for those with primary plasma cell leukemia. The median overall survival (OS) was 38 months (95% CI: 30-NR) for patients with 2–5% CTCs and 28 months (95% CI: 13-NR) for patients with primary plasma cell leukemia. Notably, there was no significant difference in PFS (*p* = 0.222) and OS (*p* = 0.473) between patients with 2–5% CTCs and those with primary plasma cell leukemia (Figure 3).

### 3.5. ≥ 2% CTCs conferred a primary plasma cell leukemia like prognosis

**Figure 4.**
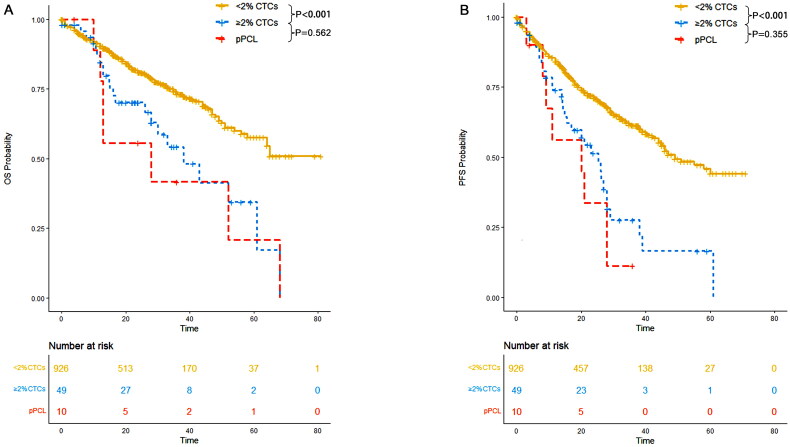
OS and PFS of patients with <2% CTCs, those with ≥2% CTCs and those with primary plasma cell leukemia. (A) OS; (B) PFS.

In our study, we compared the PFS and OS outcomes between patients with ≥2% CTCs and those with primary plasma cell leukemia. No significant difference in PFS (*p* = 0.355) and OS (*p* = 0.562) outcomes was observed between the two groups (Figure 4).

### 3.6. 1% CTCs did not confer a worse prognosis compared to those with <1% CTCs

**Figure 5.**
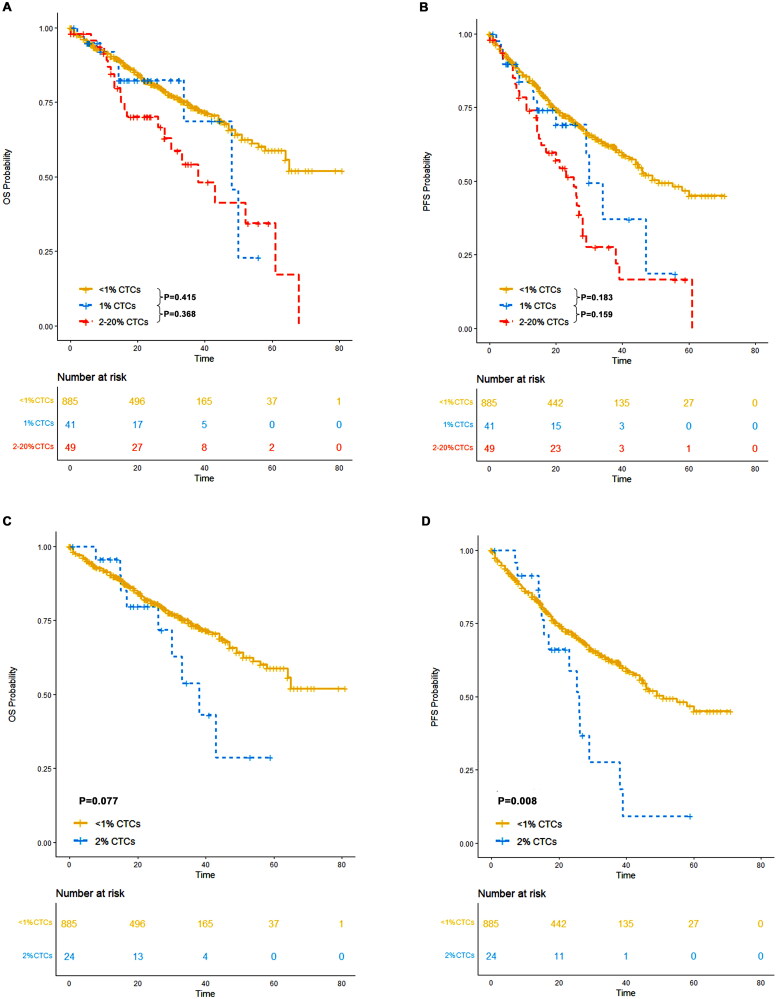
OS and PFS of patients with <1% CTCs, those with 1% CTCs, those with 2% CTCs and those with ≥2% CTCs. (A) OS of patients with <1% CTCs, those with 1% CTCs those with ≥2% CTCs; (B) PFS OS of patients with <1% CTCs, those with 1% CTCs those with ≥2% CTCs; (C) OS of patients with <1% CTCs and those with 2% CTCs; (D) PFS of patients with <1% CTCs and those with 2% CTCs.

In our study, 885 patients had <1% CTCs, 41 patients had 1% CTCs, and 49 patients had ≥2% CTCs. The median progression-free survival (PFS) was 51 months (95% CI: 45-NR) for patients with <1% CTCs, 30 months (95% CI: 29-NR) for patients with 1% CTCs, and 25 months (95% CI: 16–29) for patients with ≥2% CTCs. The median OS was not reached (95% CI: 64-NR) for patients with <1% CTCs, 48 months (95% CI: 34-NR) for those with 1% CTCs, and 38 months (95% CI: 28-NR) for patients with ≥2% CTCs. A significant difference in OS (*p* = 0.415) and PFS (*p* = 0.183) was not observed between patients with 1% CTCs and those with <1% CTCs. However, patients with 2% CTCs had a significantly worse survival outcome compared to those with <1% CTCs (Figure 5).

### 3.7. CD38 monoclonal antibody-based induction therapy and single ASCT were effective for patients with <2% CTCs but partially effective for those with ≥2% CTCs. However, daratumumab-based quadruplet therapy may be a promising option for patients with ≥2% CTCs

Induction treatments were categorized into three groups: immunomodulatory agents (IMiDs) plus proteasome inhibitors (PIs) (e.g. VRD, VTD, IRD), CD38 monoclonal antibody-based regimens (e.g. DVRD, DRD), and IMiDs or PI-based regimens (e.g. PAD, VCD).

**Figure 6.**
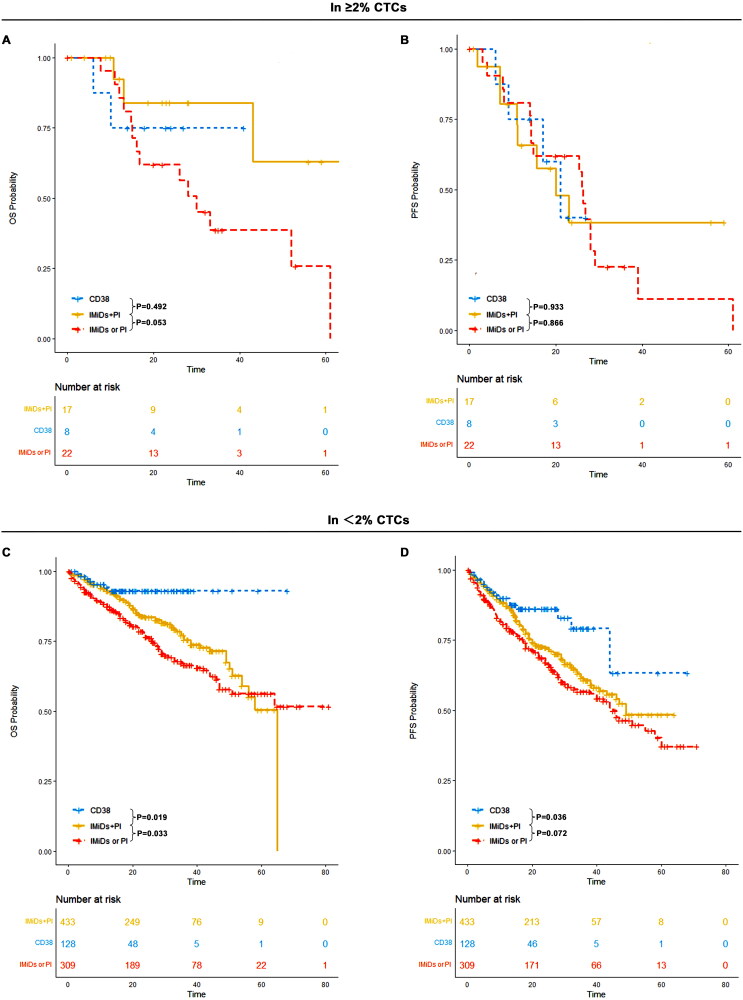
OS and PFS of patients in IMiDs plus PI, CD38 monoclonal antibody and IMiDs or PI based induction treatment in ≥2% CTCs and <2% CTCs groups. (A) OS of patients in IMiDs plus PI, CD38 monoclonal antibody and IMiDs or PI based induction treatment in ≥2% CTCs; (B) PFS of patients in IMiDs plus PI, CD38 monoclonal antibody and IMiDs or PI based induction treatment in ≥2% CTCs; (C) OS of patients in IMiDs plus PI, CD38 monoclonal antibody and IMiDs or PI based induction treatment in <2% CTCs; (D) PFS of patients in IMiDs plus PI, CD38 monoclonal antibody and IMiDs or PI based induction treatment in <2% CTCs.

In patients with <2% CTCs, the median PFS was 49 months (95% CI: 43-NR) for IMiDs plus PI, not reached (95% CI: 44-NR) for CD38, and 45 months (95% CI: 38–60) for IMiDs or PI. The median OS was 65 months (95% CI: 54-NR) for IMiDs plus PI, not reached (95% CI: NR-NR) for CD38, and not reached (95% CI: 51-NR) for IMiDs or PI alone. Patients in the CD38 group demonstrated significantly better OS and PFS compared to those in the IMiDs plus PI or IMiDs/PI-only groups (*p* < 0.05). In contrast, for patients with ≥2% CTCs, The median PFS was 20 months (95% CI: 11-NR) for IMiDs plus PI, 21 months (95% CI: 17-NR) for CD38, and 26 months (95% CI: 14-NR) for IMiDs or PI. The median OS was 68 months (95% CI: 43-NR) for IMiDs plus PI, not reached (95% CI: NR-NR) for CD38, and 30 months (95% CI: 16-NR) for IMiDs or PI alone. Notably, no significant difference in PFS (*p* = 0.98) or OS (*p* = 0.14) outcomes was observed among the three groups in this subgroup. Among patients with ≥2% CTCs, 4 out of 8 (50%) received daratumumab-based quadruplet therapy, all achieving at least very good partial response (≥VGPR) after induction. Among patients with ≥2% CTCs, 4 out of 8 (50%) received daratumumab-based quadruplet therapy, all achieving at least very good partial response (≥VGPR) after induction. Notably, only one progression was observed in this group, with a median follow-up of 23 months (95% CI: 18-NR) (Figure 6).

**Figure 7.**
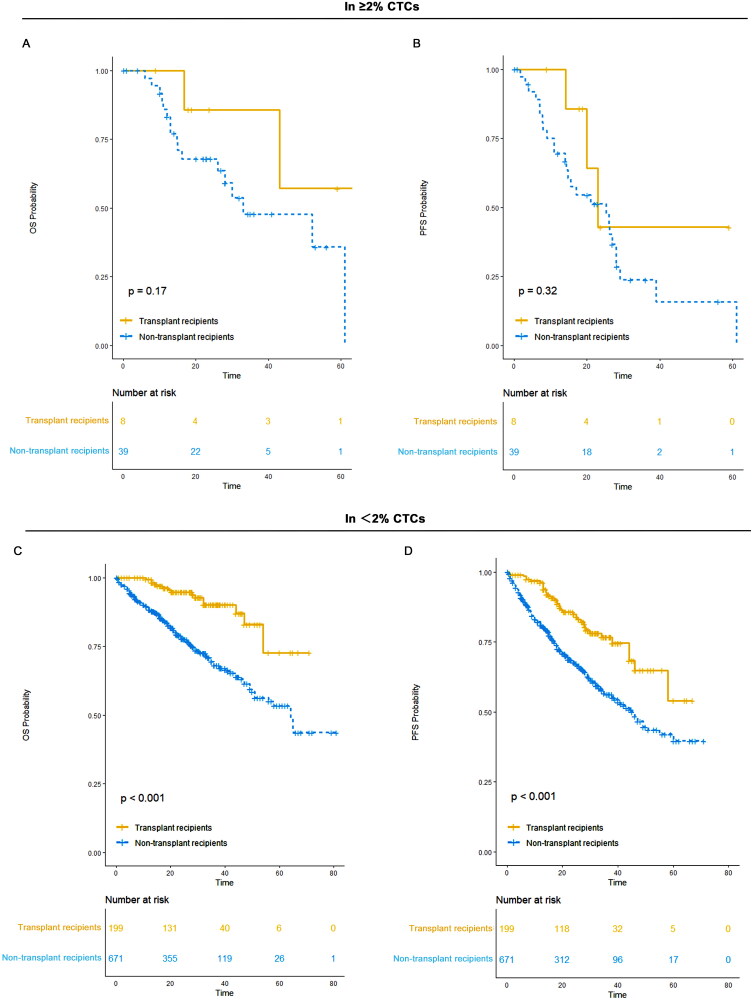
OS and PFS of transplants versus non-transplants in patients with ≥2% CTCs and those with <2% CTCs. (A) OS a of transplants versus non-transplants in patients with ≥2% CTCs; (B) PFS a of transplants versus non-transplants in patients with ≥2% CTCs; (C) OS a of transplants versus non-transplants in patients with <2% CTCs; (D) PFS of transplants versus non-transplants in patients with <2% CTCs.

In the cohort with <2% CTCs, the median PFS for transplant recipients was not reached (95% CI: 58-NR), compared to 45 months (95% CI: 39–55) for non-transplant recipients, with significant differences in PFS (*p* < 0.001) and OS (*p* < 0.001) observed between the two groups. Transplant recipients had a median OS that was not reached (95% CI: NR-NR), while non-transplant recipients had a median OS of 64 months (95% CI: 51-NR). For patients with ≥2% CTCs, the median PFS was 23 months (95% CI: 20-NR) for transplant recipients and 25 months (95% CI: 14–29) for non-transplant recipients, with no significant differences in PFS (*p* = 0.32) or OS (*p* = 0.17) or between transplant and non-transplant groups (Figure 7). The median OS was 68 months (95% CI: 43-NR) for transplant recipients and 33 months (95% CI: 26-NR) for non-transplant recipients.

### 3.8. VRD, IRD, DVRD, and DRD were reliable choices for patients with <2% CTCs, daratumumab-based quadruplet therapy may represent a promising option for patients with ≥2% CTCs

For patients with ≥2% CTCs, bortezomib, lenalidomide, and dexamethasone (VRD) was the most commonly used frontline therapy within the IMiDs plus PI group. VRD recipients achieved a median progression-free survival (PFS) of 15 months (95% CI: 11-NR) and a median OS of 68 months (95% CI: 43-NR). In the daratumumab-based therapy group, quadruplet regimens demonstrated superior efficacy, with both PFS and OS unreached (95% CI: NR-NR).

**Figure 8.**
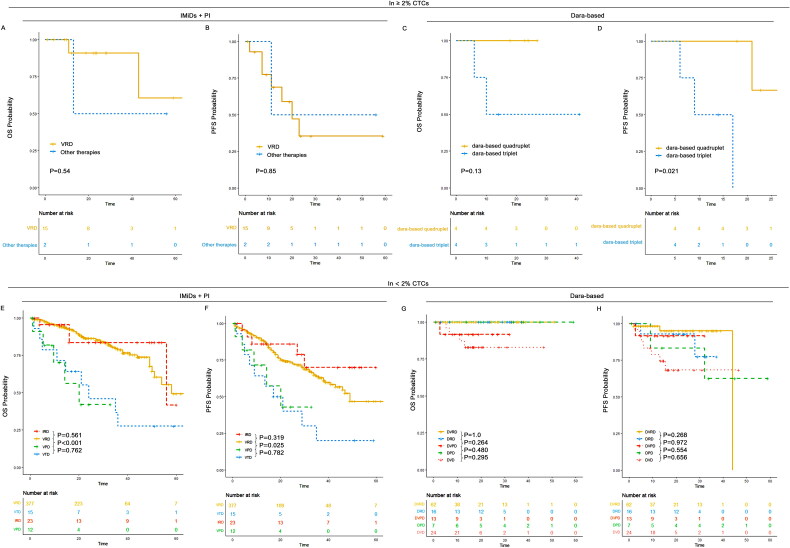
OS and PFS of specific therapy in each group. (A) OS of VRD and other therapies in patients with ≥2% CTCs; (B) PFS of VRD and other therapies in patients with ≥2% CTCs; (C) OS of daratumumab based quadruplet therapy versus daratumumab based triplet therapy in patients with ≥2% CTCs; (D) PFS of daratumumab based quadruplet therapy versus daratumumab based triplet therapy in patients with ≥2% CTCs. (E) OS of VRD, VTD, IRD and VPD in patients with <2% CTCs; (F) PFS of VRD, VTD, IRD and VPD in patients with <2% CTCs; (G) OS of DVRD, DRD, DVPD, DPD and DVD in patients with <2% CTCs; (H) PFS of DVRD, DRD, DVPD, DPD and DVD in patients with <2% CTCs.

For patients with <2% CTCs, VRD and IRD were more commonly used within the IMiDs plus PI group, showing promising survival outcomes. VRD recipients achieved a median PFS of 49 months (95% CI: 43-NR) and a median OS of 58 months (95% CI: 51-NR). IRD recipients had an unreached PFS (95% CI: NR-NR) and a median OS of 56 months (95% CI: 56-NR). In the daratumumab-based therapy group, DVRD and DRD demonstrated notable efficacy. DVRD recipients achieved an unreached median PFS (95% CI: NR-NR) and a median OS of 44 months (95% CI: NR-NR). DRD recipients achieved both an unreached median PFS and OS (95% CI: NR-NR) (Figure 8; Table 3).

**Table 3.** The median PFS and OS of different induction regimens.

≥2% CTCs		
Induction regimen	Median PFS	Median OS
Daratumumab-based quadruplet	NR (95%CI: 21-NR)	NR (95%CI: NR-NR)
Daratumumab-based triplet	13 months (95%CI: 6-NR)	10 months (95%CI: 6-NR)
VRD	15 months (95% CI: 11-NR)	68 months (95% CI: 43-NR)
DVRD	44 months (95%CI: NR-NR)	NR (95%CI: NR-NR)
DRD	NR (95%CI: NR-NR)	NR (95%CI: NR-NR)
DVPD	NR (95%CI: NR-NR)	NR (95%CI: NR-NR)
DPD	NR (95%CI: 32-NR)	NR (95%CI: NR-NR)
DVD	NR (95%CI: NR-NR)	NR (95%CI: NR-NR)
IRD	NR (95%CI: NR-NR)	56 months (95%CI: 56-NR)
VRD	49 months (95%CI: 43-NR)	58 months (95%CI: 51-NR)
VPD	20 months (95%CI: 9-NR)	20 months (95%CI: 10-NR)
VTD	19 months (95%CI: 9-NR)	24 months (95%CI: 15-NR)

VRD: bortezomib, lenalidomide, and dexamethasone; DVRD; daratumumab, bortezomib, lenalidomide, and dexamethasone; DRD: daratumumab, lenalidomide, and dexamethasone; DVPD: daratumumab, bortezomib, pomalidomide, and dexamethasone; DPD: daratumumab pomalidomide, and dexamethasone; DVD: daratumumab, bortezomib and dexamethasone; IRD: Ixazomib, lenalidomide, and dexamethasone; VPD: bortezomib, pomalidomide, and dexamethasone; VTD: bortezomib, thalidomide, and dexamethasone.

### 3.9. Achieving CR after induction could not overcome the poor prognosis conferred by ≥2% CTCs

In patients with ≥2% CTCs, 15 achieved CR or better, while 25 achieved VGPR or worse following induction treatment. Those who achieved ≥ CR had a median PFS of 25 months (95% CI: 16-NR) and an unreached median OS (95% CI: 33-NR). For patients who achieved < CR, the median PFS was 20 months (95% CI: 11-NR) and the median OS was 52 months (95% CI: 16-NR). There was no significant difference in OS (*p* = 0.18) or PFS (*p* = 0.22) between patients who achieved ≥ CR and those who achieved < CR.

**Figure 9.**
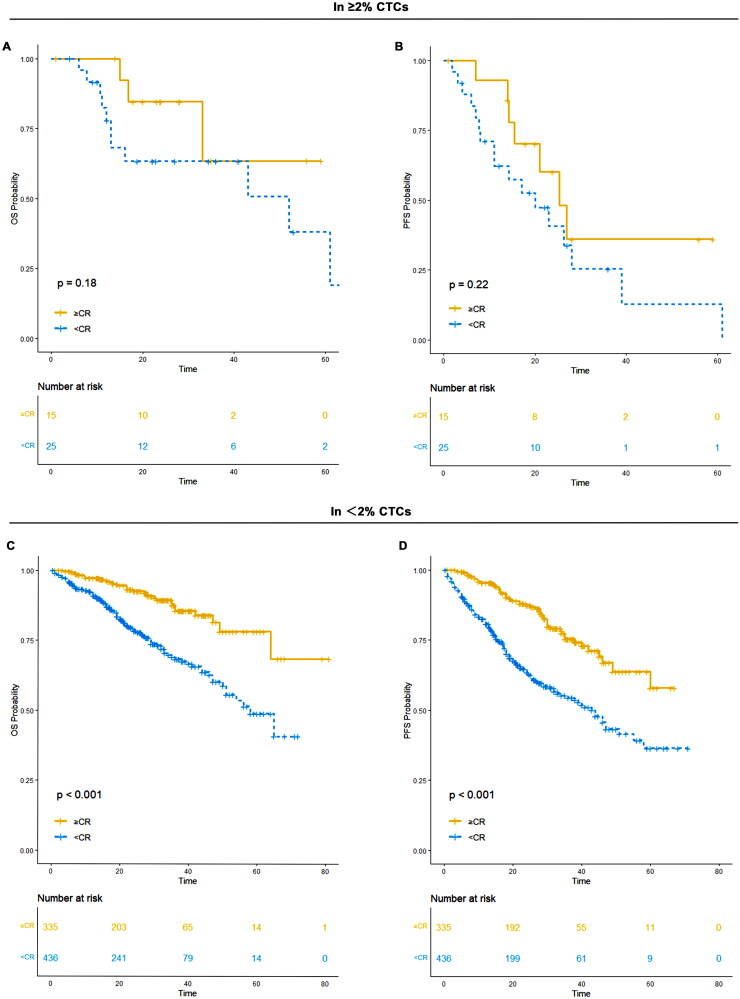
OS and PFS of patients who achieved CR/sCR to induction treatment versus those who did not in ≥2% CTCs and <2% CTCs group. (A) OS of patients who achieved CR/sCR to induction treatment versus those who did not in ≥2% CTCs; (B) PFS of patients who achieved CR/sCR to induction treatment versus those who did not in ≥2% CTCs; (C) OS of patients who achieved CR/sCR to induction treatment versus those who did not in <2% CTCs; (D) PFS of patients who achieved CR/sCR to induction treatment versus those who did not in <2% CTCs.

Among patients with <2% CTCs, 335 achieved CR or better, and 436 achieved VGPR or worse. Patients who reached ≥ CR had both an unreached median PFS (95% CI: 60-NR) and an unreached median OS (95% CI: NR-NR). In contrast, those who achieved < CR had a median PFS of 43 months (95% CI: 34–55) and a median OS of 58 months (95% CI: 51-NR). Achieving ≥ CR conferred significantly improved PFS (*p* < 0.001) and OS (*p* < 0.001) compared to achieving < CR (Table 4; Figure 9).

**Table 4.** Response to induction treatment in <2% CTCs and ≥2% CTCs.

Response (n/%)	<2% CTCs	≥2% CTCs	*p* Value
sCR/CR	335 (43.5)	15 (37.5)	0.553
VGPR	193 (25.0)	9 (22.5)	
PR	172 (22.3)	9 (22.5)	
MR	18 (2.3)	1 (2.5)	
SD	42 (5.4)	5 (12.5)	
PD	11 (1.4)	1 (2.5)	

CR: complete remission; VGPR: very good partial remission; PR: partial remission; MR: minimal residual disease; SD: stable disease; PD: progressive disease.

### 3.10. 1 HRA worsens outcomes in <2% CTCs, while ≥2 HRA worsens outcomes in ≥2% CTCs

For patients with <2% CTCs, the median PFS was 58 months (95% CI: 47-NR) for patients without HRA, 34 months (95% CI: 29–46) for those with 1 HRA, and 25 months (95% CI: 19–36) for those with ≥2 HRA. The median OS was not reached (95% CI: 65-NR) for those without HRA, 50 months (95% CI: 44-NR) for patients with 1 HRA, and 32 months (95% CI: 26-NR) for those with ≥2 HRA. Patients with 1 HRA had a significantly worse PFS (*p* < 0.001) and OS (*p* < 0.001) compared to those without HRA. Furthermore, patients with ≥2 HRA had significantly worse PFS (*p* = 0.04) compared to those with 1 HRA.

**Figure 10.**
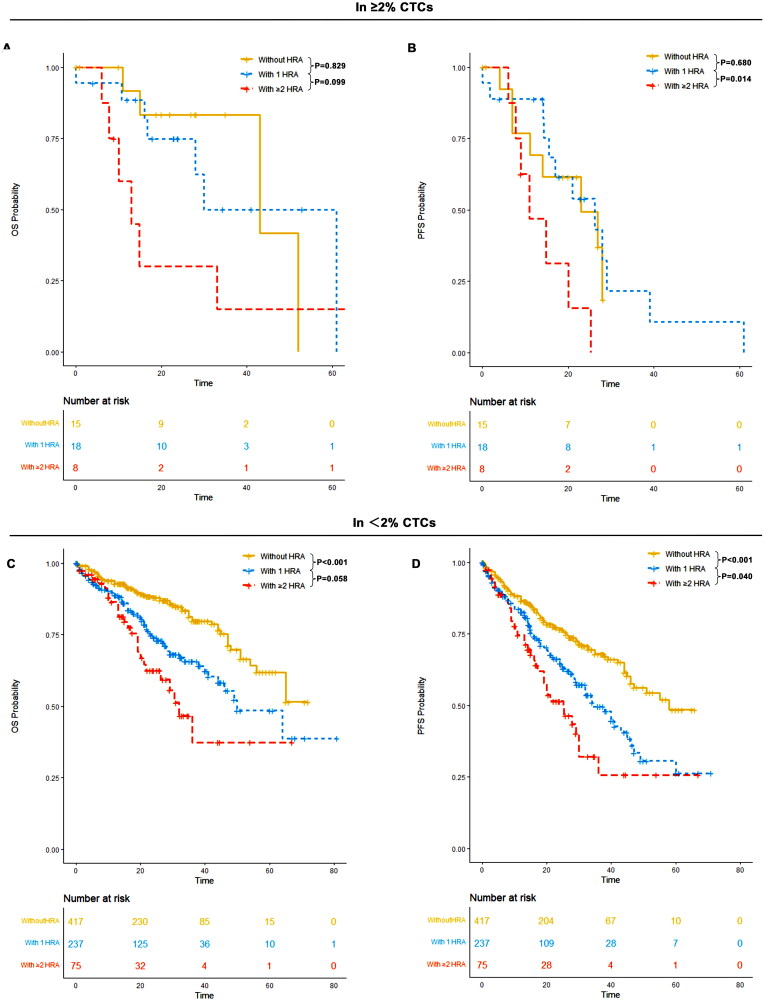
OS and PFS of patients without HRA, those with 1 HRA and those with ≥2 HRA in ≥2% CTCs and <2% CTCs groups. (A) OS patients without HRA, those with 1 HRA and those with ≥2 HRA in ≥2% CTCs; (B) PFS of patients without HRA, those with 1 HRA and those with ≥2 HRA in ≥2% CTCs; (C) OS patients without HRA, those with 1 HRA and those with ≥2 HRA in <2% CTCs; (D) PFS of patients without HRA, those with 1 HRA and those with ≥2 HRA in <2% CTCs.

For patients with ≥2% CTCs, the median PFS was 23 months (95% CI: 11-NR) for those without HRA, 26 months (95% CI: 16-NR) for patients with 1 HRA, and 11 months (95% CI: 9-NR) for those with ≥2 HRA. The median OS was 43 months (95% CI: 43-NR) for those without HRA, 30 months (95% CI: 28-NR) for patients with 1 HRA, and 13 months (95% CI: 10-NR) for those with ≥2 HRA. No significant difference in PFS (*p* = 0.680) and OS (*p* = 0.829) was observed between patients with 1 HRA and those without HRA. However, patients with ≥2 HRA had significantly worse PFS and OS outcomes compared to those without HRA and those with only 1 HRA (Figure 10).

### 3.11. Concomitant 1q21+ and other HRA conferred a worse prognosis

For patients with <2% CTCs, the median PFS was 34 months (95% CI: 29–45) for patients with 1q21+ only, 19 months (95% CI: 10-NR) for patients with 1q21+ plus 17p-, and 28 months (95% CI: 16-NR) for patients with 1q21+ plus t(4;14). The median OS was 50 months (95% CI: 41-NR) for those with 1q21+ only, 32 months (95% CI: 19-NR) for those with 1q21+ plus 17p-, and not reached (95% CI: 29-NR) for those with 1q21+ plus t(4;14). Patients with 1q21+ only, 1q21+ plus 17p-, and 1q21+ plus t(4;14) had significantly worse OS and PFS outcomes compared to those without HRA. A significant difference in OS and PFS outcomes was also observed between those without HRA and those with 1q21+ only. Notably, patients with 1q21+ plus 17p- exhibited significantly worse OS (*p* = 0.008) and PFS (*p* = 0.005) compared to those with 1q21+ only.

**Figure 11.**
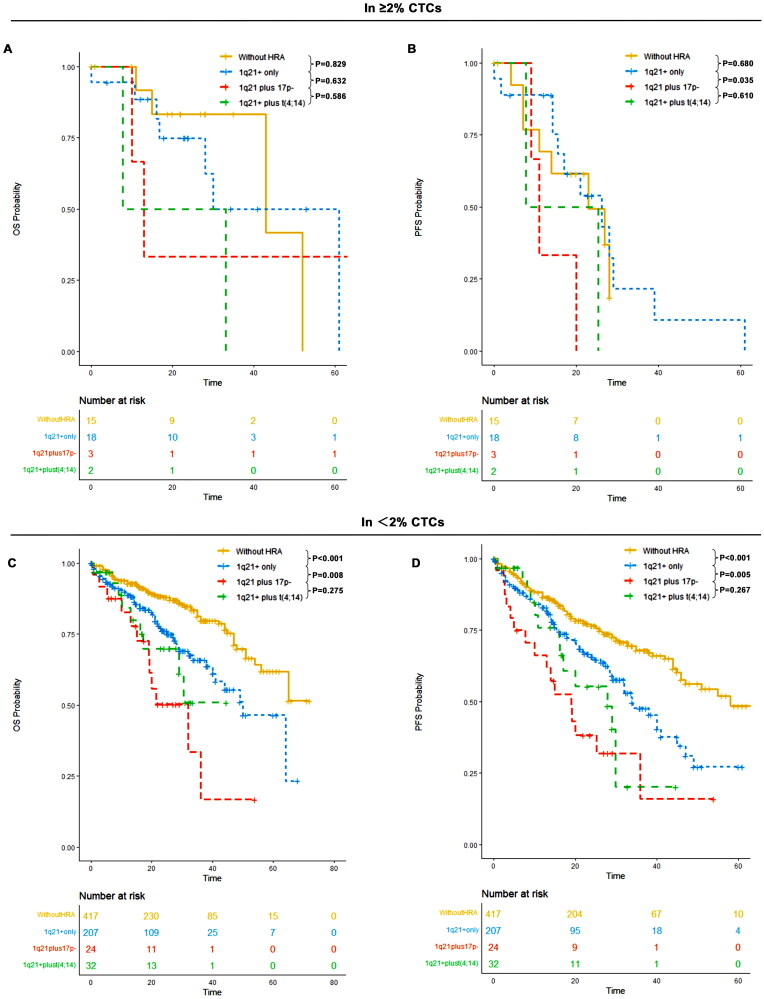
OS and PFS of patients without HRA, those with 1q21+ only, those with 1q21+ plus 17p- and those with 1q21+ plus t(4;14) in ≥2% CTCs and <2% CTCs groups. (A) OS of patients without HRA, those with 1q21+ only, those with 1q21+ plus 17p- and those with 1q21+ plus t(4;14) in ≥2% CTCs; (B) PFS of patients without HRA, those with 1q21+ only, those with 1q21+ plus 17p- and those with 1q21+ plus t(4;14) in ≥2% CTCs; (C) OS of patients without HRA, those with 1q21+ only, those with 1q21+ plus 17p- and those with 1q21+ plus t(4;14) in <2% CTCs; (D) PFS of patients without HRA, those with 1q21+ only, those with 1q21+ plus 17p- and those with 1q21+ plus t(4;14) in <2% CTCs.

For patients with ≥2% CTCs, the median PFS was 26 months (95% CI: 16-NR) for patients with 1q21+ only, 11 months (95% CI: 9-NR) for those with 1q21+ plus 17p-, and 17 months (95% CI: 8-NR) for those with 1q21+ plus t(4;14). The median OS was 30 months (95% CI: 28-NR) for those with 1q21+ only, 13 months (95% CI: 10-NR) for those with 1q21+ plus 17p-, and 20 months (95% CI: 8-NR) for those with 1q21+ plus t(4;14). Patients with 1q21+ plus t(4;14) had a significantly worse OS compared to those without HRA. However, no significant difference in OS or PFS was observed between those without HRA and those with 1q21+ only. Additionally, patients with 1q21+ plus 17p- showed significantly worse PFS (*p* = 0.035) compared to those with 1q21+ only (Figure 11).

### 3.12. De novo EME may represent a distinct entity within multiple myeloma

**Figure 12.**
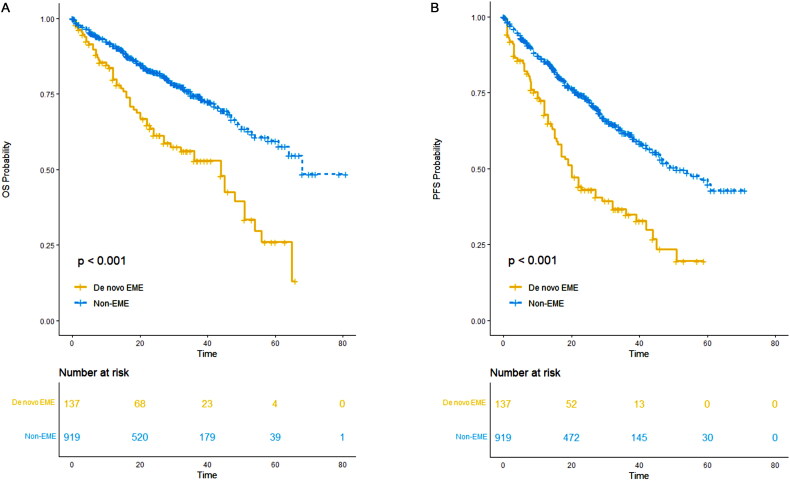
OS and PFS of patients with de novo EME versus those with non-EME. (A) OS of patients with de novo EME versus those with non-EME; (B) PFS of patients with de novo EME versus those with non-EME.

In our study, 137 patients presented with de novo EME, while 919 patients had non-EME, which included both EMB and non-EMD subtypes. Patients with de novo EME demonstrated a median PFS of 20 months (95% CI: 16–32) and a median OS of 44 months (95% CI: 27–51). In contrast, patients with non-EME exhibited a median PFS of 51 months (95% CI: 46-NR) and a median OS of 68 months (95% CI: 64-NR). The difference in PFS (*p* < 0.001) and OS (*p* < 0.001) between de novo EME and non-EME groups was significant. Findings from our study, combined with data from previous research, suggest that de novo EME may represent a distinct clinical entity within multiple myeloma, warranting dedicated consideration in both clinical practice and research [17–19] (Figure 12).

### 3.13. ≥ 2% CTCs was a predictor of poor prognosis in de novo EME and non-EME

In patients with de novo EME, those with ≥2% CTCs had a median PFS of 7 months (95% CI: 3-NR) and a median OS of 11 months (95% CI: 8-NR). In contrast, patients with <2% CTCs had a median PFS of 20 months (95% CI: 16–36) and a median OS of 45 months (95% CI: 36–56). This difference in PFS (*p* = 0.013) and OS (*p* = 0.002) between the two groups was statistically significant.

**Figure 13.**
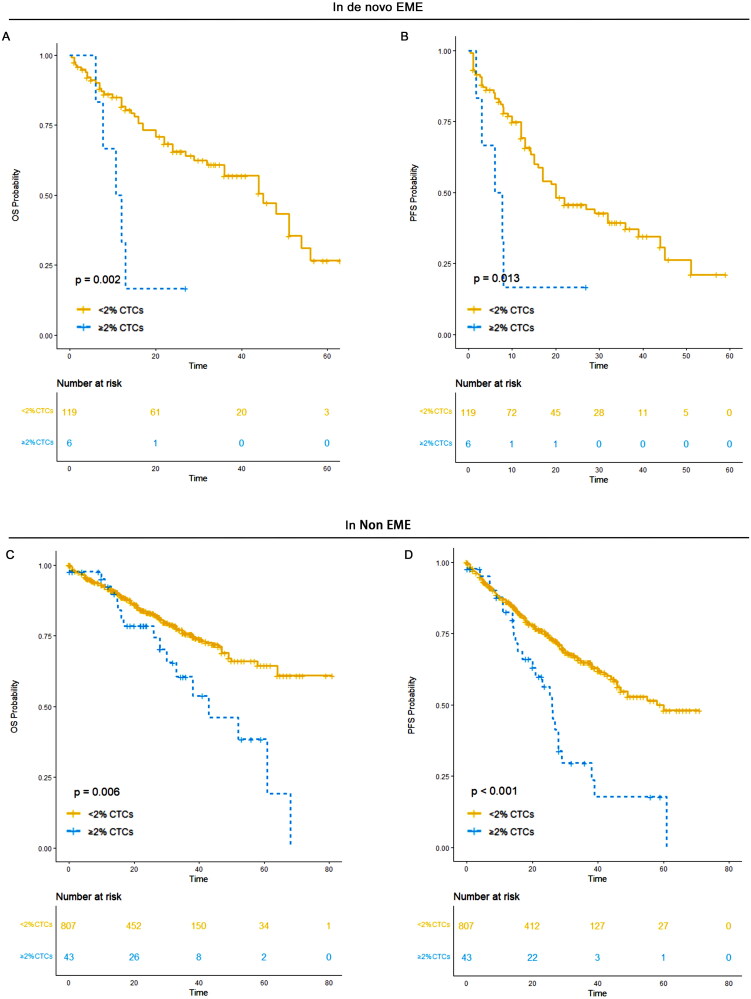
OS and PFS of ≥2% CTCs versus <2% CTCs in de novo EME and non-EME groups. (A) OS a of ≥2% CTCs versus <2% CTCs in de novo EME; (B) PFS a of ≥2% CTCs versus <2% CTCs in de novo EME; (C) OS a of ≥2% CTCs versus <2% CTCs in Non-EME; (D) PFS a of ≥2% CTCs versus <2% CTCs in Non-EME.

In non-EME patients, those with ≥2% CTCs demonstrated a median PFS of 26 months (95% CI: 20–38) and a median OS of 43 months (95% CI: 33-NR). For patients with <2% CTCs, the median PFS was 58 months (95% CI: 47-NR) and the median OS was unreached (95% CI: NR-NR). Significant differences in PFS (*p* < 0.001) and OS (*p* = 0.006) were observed between the two CTC groups in non-EME patients (Figure 13).

### 3.14. Multivariable analysis

**Figure 14.**
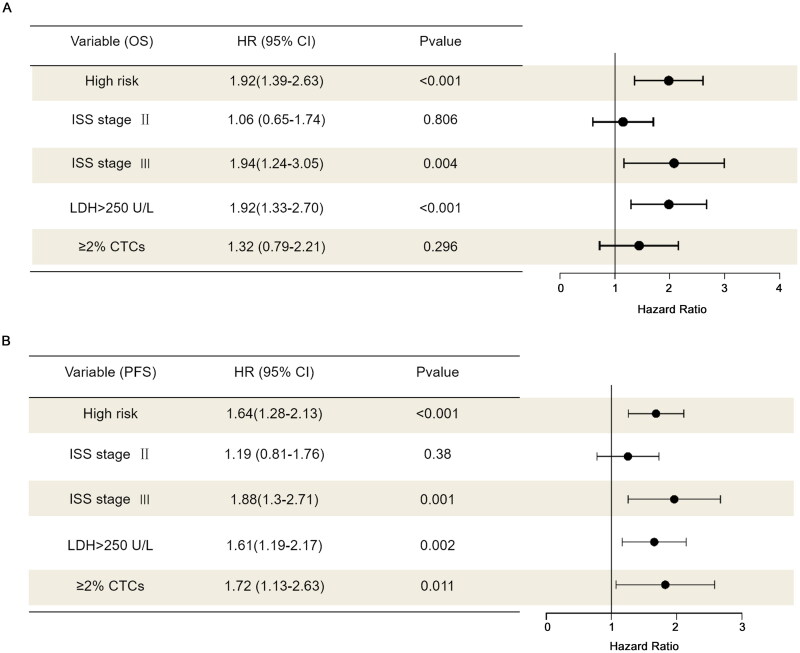
Multivariable analysis of OS and PFS for the entire cohort. (A) OS; (B) PFS.

In our study, ISS stage, cytogenetic risk stratification, LDH level, and CTC levels were included in the multivariable analysis. ≥2% CTCs was identified as an independent factor associated with worse PFS outcome (*p* = 0.011) but was not independently associated with worse OS outcome (*p* = 0.296) (Figure 14).

## 4. Discussion

In this study, we included 1,056 patients with NDMM to evaluate the clinical relevance of a 2% CTCs threshold. Patients with ≥2% CTCs were classified as an ultra-high-risk multiple myeloma population, exhibiting plasma cell leukemia (pPCL)-like prognoses. Our objective is to assess the significance of this cutoff within a large multiple myeloma database in China.

In our study, patients with ≥2% CTCs showed significantly worse PFS (*p* < 0.001; 49 months versus 25 months) and OS (*p* < 0.001; NR versus 38 months) compared to those with <2% CTCs. These shorter PFS and OS outcomes provide some evidence that 2% CTCs might serve as an indicator of ultra-high-risk multiple myeloma patients. In the largest published dataset on primary plasma cell leukemia, the median OS was reported as 23 months (95% CI: 15–34), a survival duration comparable to that of patients with ≥2% CTCs in the era of novel agents [20]. In a 2011 multicenter study involving 73 patients with primary plasma cell leukemia, the median OS was only 12.6 months [10]. In a 2015 study, patients with more than 2% CTCs demonstrated comparable PFS and OS to those with primary plasma cell leukemia, with a median PFS of 12 months and a median OS of 15 months [21]. The clear difference in OS for primary plasma cell leukemia between the eras of conventional therapy and novel agents highlights the critical impact of advancing treatments [2,22–24]. Looking ahead, patients with primary plasma cell leukemia may achieve longer survival outcomes as more effective therapies are developed and implemented. Additionally, we observed that patients with ≥2% CTCs and those meeting pPCL criteria had similar outcomes, suggesting that 2% could serve as a potential threshold for identifying a high-risk group. To further explore this, we examined the PFS and OS between patients with 1% CTCs and those with <1% CTCs, finding no significant differences in these outcomes. This may suggest that 1% CTCs may not reliably identify ultra-high-risk MM, and that 2% could potentially serve as a threshold for identifying this high-risk population, although further investigation is required to confirm its validity.

In recent years, CTCs have also emerged as a reliable biomarker, especially when combined with other factors such as RISS stage or FDG PET/CT, to enhance risk stratification in multiple myeloma. These studies provide robust support for the clinical application of CTCs in managing and accessing risk in myeloma patients [25–28].

After establishing that 2% CTCs may be a reliable cutoff for defining an ultra-high-risk population, we examined whether daratumumab-based therapy and autologous stem cell transplantation (ASCT) could provide effective treatment options for patients with ≥2% CTCs, given the superior efficacy of daratumumab observed in both clinical trials and real-world settings. Our prior findings also supported this efficacy. However, in patients with ≥2% CTCs, daratumumab-based frontline therapy did not confer improved survival outcomes compared to other induction regimens. In a multicenter study conducted in 2023, survival outcomes improved for patients receiving daratumumab-based quadruplet therapy [29]. In our study, 4 out of 8 patients (50%) received daratumumab-based quadruplet therapy, all achieving ≥ VGPR (very good partial response) after induction, with no cases of progression or death. The median follow-up for these patients was 23 months (95% CI: 18-NR). These findings align with the improved prognosis observed in the multicenter study conducted in 2023 for patients on daratumumab-based quadruplet therapy. Daratumumab-based quadruplet therapy may be a promising option for patients with ≥2% CTCs. Our analysis further examined specific therapeutic regimens for patients based on CTC levels. The results indicated that VRD and IRD were reliable choices for patients with <2% CTCs within the IMiDs plus PI group, while DVRD and DRD remained dependable options in the CD38 monoclonal antibody-based group. For patients with ≥2% CTCs, daratumumab-based quadruplet therapy demonstrated promising effectiveness, suggesting it may be a reliable treatment option for this high-risk subgroup. Additionally, single ASCT showed effectiveness on improving survival outcomes in this high-risk group. This suggests that using daratumumab only as a frontline therapy may be insufficient for patients with ≥2% CTCs; extending daratumumab use into maintenance treatment might enhance outcomes. Moreover, tandem ASCT may offer more benefit for this ultra-high-risk subset. In contrast, for patients with <2% CTCs, both daratumumab-based therapy and single ASCT significantly improved OS and PFS. For patients with ≥2% CTCs, more effective treatment strategies are still needed. Future studies may need to investigate the use of immunotherapies for patients with ≥2% CTCs.

Since our analysis demonstrated that ≥2% CTCs and <2% CTCs represent distinct prognostic groups, we further investigated the impact of the number of high-risk abnormalities (HRA) on survival outcomes within each group. In the ≥2% CTCs group, patients with ≥2 HRA were identified as having significantly poorer survival outcomes, whereas the presence of only 1 HRA did not markedly impact survival.

The gain or amplification of 1q21 is the most frequently observed high-risk cytogenetic abnormality (HRA) in multiple myeloma, with numerous studies assessing its impact in real-world settings [30–32]. However, whether 1q21+ alone should be categorized as an HRA remains contentious. In our study, we analyzed survival outcomes for patients with 1q21+ alone compared to those with 1q21+ accompanied by other HRA. For patients with ≥2% CTCs, 1q21+ alone did not confer a survival advantage over those without HRA, while patients with 1q21+ plus 17p- had significantly shorter survival than those with 1q21+ alone or no HRA. In contrast, among patients with <2% CTCs, 1q21+ alone was associated with significantly worse PFS and OS compared to patients without HRA. Furthermore, patients with 1q21+ plus 17p- had shorter PFS and OS than those with 1q21+ alone. These findings indicate that the impact of 1q21+ on survival varies significantly between the ≥2% CTCs and <2% CTCs groups, suggesting distinct characteristics and implications for each population.

De novo EME is also considered an ultra-high-risk population in multiple myeloma [18,19,33]. We examined survival outcomes based on CTC levels in both de novo EME and Non-EME groups, comparing those with ≥2% CTCs versus <2% CTCs. In both groups, a threshold of 2% CTCs served as a robust predictor for identifying patients with significantly worse survival outcomes, underscoring its clinical relevance as a marker of ultra-high-risk disease.

In the multivariable analysis for PFS, ≥2% CTCs remained a significant factor associated with shorter PFS outcomes, but this variable lost its significance in the multivariable analysis for OS. We hypothesize that this discrepancy may be attributed to three key aspects. First, OS outcomes are influenced by a broader range of factors, such as salvage treatments after disease progression, comorbidities, and other non-disease-related causes of death, which may diminish the apparent effect of ≥2% CTCs. Second, PFS events (progression or death) occur more frequently than OS events, providing greater statistical power in PFS analyses, whereas the smaller number of OS events may reduce the ability to detect significant associations. Third, the independent effect of ≥2% CTCs may be diluted by other variables included in the multivariable model, particularly those with stronger impacts on OS.

Our study systematically evaluated the applicability of a 2% CTC threshold in a real-world setting using a large NDMM database in the era of novel agents, effectively validating the prognostic value of ≥2% CTCs. However, certain limitations remain. First, as a retrospective study, there is potential for inherent bias. Additionally, the relatively small number of patients who received daratumumab-based maintenance therapy or tandem ASCT limited our ability to fully explore optimal treatments for this ultra-high-risk population.

**Figure 15.**
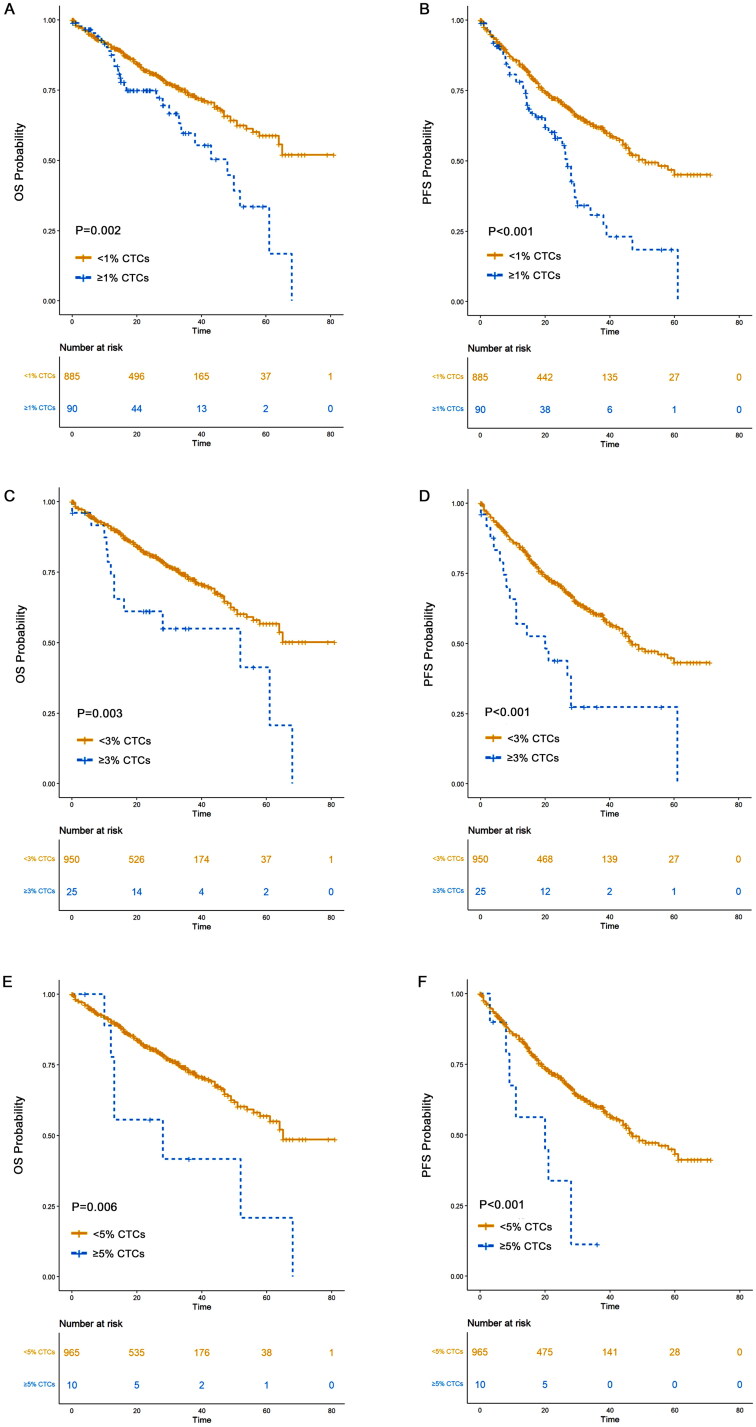
OS and PFS of patients with different CTCs percentage. (A) OS of patients with <1% CTCs versus those with ≥1% CTCs; (B) PFS of patients with <1% CTCs versus those with ≥1% CTCs; (C) OS of patients with <3% CTCs versus those with ≥3% CTCs; (D) PFS of patients with <3% CTCs versus those with ≥3% CTCs; (E) OS of patients with <5% CTCs versus those with ≥5% CTCs; (F) PFS of patients with <5% CTCs versus those with ≥5% CTCs.

**Figure 16.**
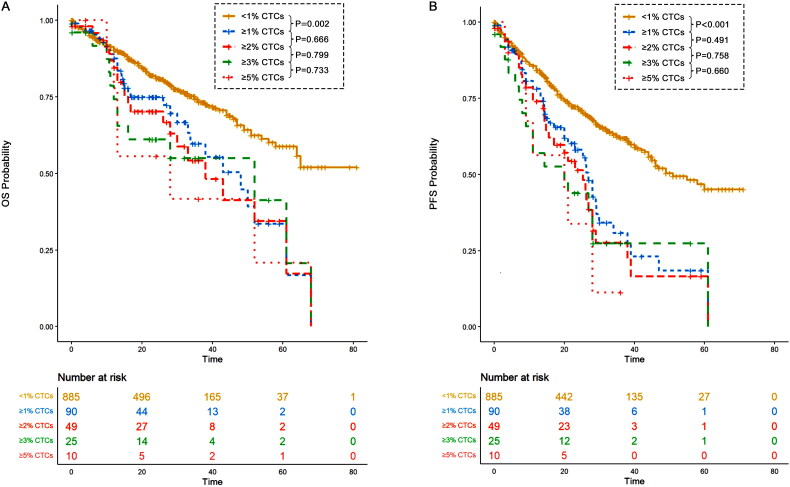
OS and PFS of patients with different CTCs percentage. (A) OS of patients with <1% CTCs, those with ≥1% CTCs, those with ≥2% CTCs, those with ≥3% CTCs and those with ≥5% CTCs; (B) PFS of patients with <1% CTCs, those with ≥1% CTCs, those with ≥2% CTCs, those with ≥3% CTCs and those with ≥5% CTCs.

In our study, we aimed to evaluate a 2% CTC cutoff as a criterion to identify multiple myeloma patients with a prognosis similar to pPCL. However, the limited number of patients with ≥3% or ≥5% CTCs restricted our statistical power to definitively determine the optimal cutoff for pPCL. As a result, we focused primarily on the 2% CTC cutoff, which represents a limitation of our study. To enhance the analysis, we also examined additional cutoffs, including 1%, 3%, and 5%, to better understand their potential prognostic implications (Figures 15 and 16). Additionally, a meaningful comparison between the 2–5% and ≥5% cohorts cannot be made due to the insufficient sample size and imbalance. Therefore, this comparison should be interpreted with caution.

In our study, flow cytometric analysis of CTCs was not routinely performed, and the subjective nature of morphologic assessment raises concerns about the accuracy of investigating low percentages of CTCs. Consequently, the adverse effect of low percentages of CTCs could not be thoroughly explored, which represents a major limitation of our study. Therefore, the results related to this aspect in our manuscript should be interpreted with caution.

In a study published in *Haematologica*, no significant statistical difference was observed between patients with 1–4% CTCs and those with 0% CTCs [14]. That study included patients treated between January 2008 and December 2013, whereas our study focused on patients enrolled between January 2016 and October 2023. Additionally, the induction treatments in the *Haematologica* study were primarily based on IMiDs, PIs, or alkylating agents. In contrast, our study involved more advanced therapies, including IMiDs combined with PIs and daratumumab-based regimens. Both studies employed Wright-Giemsa-stained peripheral blood smears to evaluate CTC counts. A recent study in the *Journal of Clinical Oncology* identified ≥0.01% CTCs as a novel risk factor for transplant-eligible patients [26]. This underscores the growing recognition of the adverse prognostic impact of even small CTC percentages in the current treatment era, compared to earlier studies. We hypothesize that advancements in multiple myeloma therapies may have led to an evolutionary increase in CTC aggressiveness, which may explain the association between even minimal CTC levels and poorer survival outcomes. This increase in aggressiveness might also account for the discrepancy between the findings of the study published in *Haematologica* and our study.

Our study suggests that *a* ≥ 2% CTC threshold may help identify an ultra-high-risk subset within multiple myeloma, highlighting the potential need for distinct treatment strategies and further investigation for this population. Importantly, our objective is not to establish new criteria for defining primary plasma cell leukemia but rather to identify an ultra-high-risk subset that could enhance clinical assessment and inform treatment strategies in practice.

## Data Availability

Data can be obtained from the corresponding author upon reasonable request.
